# BdcA, a Protein Important for *Escherichia coli* Biofilm Dispersal, Is a Short-Chain Dehydrogenase/Reductase that Binds Specifically to NADPH

**DOI:** 10.1371/journal.pone.0105751

**Published:** 2014-09-22

**Authors:** Dana M. Lord, Ayse Uzgoren Baran, Thomas K. Wood, Wolfgang Peti, Rebecca Page

**Affiliations:** 1 Department of Molecular Biology, Cell Biology and Biochemistry, Brown University, Providence, Rhode Island, United States of America; 2 Graduate Program in Molecular Pharmacology and Physiology, Brown University, Providence, Rhode Island, United States of America; 3 Department of Molecular Pharmacology, Physiology and Biotechnology and Department of Chemistry, Brown University, Providence, Rhode Island, United States of America; 4 Department of Chemical Engineering and Department of Biochemistry and Molecular Biology, Pennsylvania State University, University Park, Pennsylvania, United States of America; University of Graz, Austria

## Abstract

The *Escherichia coli* protein BdcA (previously referred to as YjgI) plays a key role in the dispersal of cells from bacterial biofilms, and its constitutive activation provides an attractive therapeutic target for dismantling these communities. In order to investigate the function of BdcA at a molecular level, we integrated structural and functional studies. Our 2.05 Å structure of BdcA shows that it is a member of the NAD(P)(H)-dependent short-chain dehydrogenase/reductase (SDR) superfamily. Structural comparisons with other members of the SDR family suggested that BdcA binds NADP(H). This was demonstrated experimentally using thermal denaturation studies, which showed that BcdA binds specifically to NADPH. Subsequent ITC experiments further confirmed this result and reported a K_d_ of 25.9 µM. Thus, BdcA represents the newest member of the limited number of oxidoreductases shown to be involved in quorum sensing and biofilm dispersal.

## Introduction

Biofilms are communities of bacterial cells that are encapsulated in a self-produced polymeric matrix that can attach to almost any surface [1]. These sessile communities are responsible for 65–80% of human infections and can also cause biocorrosion and biofouling [2]–[4]. Critically, these communities are 100-1000-fold more tolerant and/or resistant to antimicrobial therapy compared to planktonic cells [5]. As planktonic cells are more vulnerable to antibiotic therapy, one strategy for inhibiting biofilm formation is to promote the dispersal of cells from the biofilm.

The gene *bdcA* (also referred to as *yjgI*) was previously identified as a regulator of biofilm dispersal. BdcA expression decreases extracellular polysaccharide (EPS) production, cell length and aggregation while simultaneously increasing extracellular DNA production and motility [6]. These are well-known phenotypes associated with decreasing c-di-GMP concentrations, and consequently biofilm dispersal [7]–[10]. The *bdcA* knockout decreases biofilm dispersal in both a static biofilm assay and a flow cell assay (3 to 6-fold, respectively) and this phenotype was complemented by expression of *bdcA* [6].

To understand the molecular role of BdcA in biofilm dispersal, we pursued structure-function studies of the *E. coli* BdcA protein. Here, we describe the crystal structure of BdcA at 2.05 Å, where we show that BdcA is a member of the short-chain dehydrogenase/reductase family. Furthermore, using isothermal titration calorimetry (ITC) and thermal stabilization assays, we identified the biologically relevant cofactor of BdcA to be NADPH. Because BdcA plays a key role in the dispersal of bacterial biofilms, this NADPH-specific oxidoreductase provides an attractive therapeutic target for disrupting these communities and, in turn, for improving health.

## Materials and Methods

### Protein Expression and Purification

The full-length *bdcA* gene from *E. coli* was sub-cloned into the RP1B bacterial expression vector, which contains an N-terminal Thio_6_-His_6_-tag and Tobacco Etch Virus (TEV) cleavage site [11]. The plasmid was transformed into *E. coli* BL21-Gold (DE3) Competent Cells (Agilent) and subsequently inoculated into 1 L cultures of LB containing 50 mg/L kanamycin. The cells were grown at 37°C (250 rpm) to an OD_600_ of 0.6, at which point the cells were transferred to 4°C for 1 hour. The cultures were induced with 0.5 mM IPTG and grown overnight at 18°C (250 rpm).

For purification, the pellets were resuspended in lysis buffer (50 mM Tris pH 8.0, 500 mM NaCl, 0.1% Triton X-100, 5 mM imidazole, Complete tablets-EDTA free [Roche]). The cells were lysed using high-pressure homogenization (C3 Emulsiflex; Avestin) and the cell debris was removed by centrifugation (45,500×g, 50 min, 4°C). The supernatant was filtered through a 0.22-μm membrane (Millipore) and loaded onto a HisTrap HP column (GE Healthcare). His_6_-tagged BdcA was eluted using a 5–500 mM imidazole gradient. The fractions containing BdcA were identified by SDS-PAGE and pooled. The His_6_-tag was removed using proteolytic cleavage by overnight incubation with TEV protease (50 mM Tris pH 8.0, 500 mM NaCl, 4°C). Cleavage was verified by SDS-PAGE. BdcA was further purified using Ni-NTA (Qiagen) to isolate cleaved protein from the TEV protease (itself His_6_-tagged) and the cleaved His_6_-tag. Untagged BdcA was purified in a final step using size exclusion chromatography (SEC; Superdex 75 26/60, GE Healthcare; SEC buffer: 20 mM Tris pH 7.5, 100 mM NaCl, 0.5 mM TCEP). To determine the oligomerization state of BdcA, the elution volume was compared to that of MW weight standards (BioRad; 158 kDa, γ-globulin; 44 kDa, ovalbumin; 17 kDa, myoglobin). The protein was concentrated to 9.5 mg/ml and either frozen and stored at −80°C or used immediately for crystallization trials.

### Crystallization, Data Collection, and Processing

BcdA was crystallized at room temperature in 50% (v/v) PEG200, 0.1 M Tris pH 7.0, 0.05 M Li_2_SO_4_ using the sitting drop vapor diffusion method (200 µL drops). A dataset was collected at the NSLS X29 beamline at a wavelength of 1.075 Å using an ADSC Q315 CCD detector. Diffraction data were processed to 2.05 Å with HKL2000 [12]. The *R. prowazekii* FabG structure (PDB 3F9I) was identified by the Fold and Function Assignment Server (−85.1 score, 33% sequence identity) as a suitable initial molecular replacement (MR) model [13], [14]. Chainsaw was used to truncate the side chains at Cβ [15] and PHASER as part of *PHENIX* was used for MR [16]. Approximately 70% of the structure was built automatically using AutoBuild. Iterative model building and refinement were performed using COOT [17] and *PHENIX* [18]. The final model was refined with *PHENIX* using TLS. MOLPROBITY was used for model validation [19]. Analysis of the dimerization interface was performed using the Protein Interaction Calculator [20], with solvent accessible surface areas calculated using NACCESS [21]. Data collection and structure refinement statistics are reported in **Table 1**.

**Table 1:** Crystal data and data-collection statistics.

	BdcA
*Data Collection*	
Space group	*C*2
Unit-cell parameters	
*a,b,c* (Å)	131.1, 52.5, 69.8
*α, β, γ (°)*	90.0, 118.1, 90.0
Resolution	50.0 – 2.05 (2.09 – 2.05)
*R*_merge_ ¥	6.4 (59.7)
<I/σ(I)>	14.3 (2.9)
Completeness (%)	99.3 (99.2)
Multiplicity	3.6 (3.7)
Unique Reflections	26365
*Refinement*	
*Rwork/Rfree*	20.1/22.2
Protein atoms	2693
Non-protein atoms	109
Waters	53
PEG	56
Mean B-factor (Å^2^)	42.0
r.m.s.d. bond length (Å)	0.002
r.m.s.d. bond angle (°)	0.591
*Ramachandran Plot*	
Favored (%)	99.7
Allowed (%)	100.0
PDBID Code	4PCV

Values in parentheses are for the highest resolution shell.

¥ *R_merge_* = Σ*_hkl_*Σ_i_ |*I*_i_(*hkl*) - <*I*(*hkl*)>|/Σ*_hkl_*Σ_i_ *I*_i_(*hkl*) where *I*_i_(*hkl*) is the i^th^ observation of a symmetry equivalent reflection hkl. *Rfree was calculated using 5% of the reflections omitted from the refinement.

### Differential Scanning Fluorimetry

SEC (Superdex 75 26/60, GE Healthcare) was used to transfer BdcA into assay buffer (20 mM HEPES pH 7.5). A series of protein thermal denaturation assays were performed, which contained a final concentration of 8 µM protein, 160 µM ligand solubilized in assay buffer, and 5× SYPRO Orange (Invitrogen). As a control, buffer was used instead of ligand. Samples were aliquoted in a 96-well PCR plate (Applied Biosystems) and sealed with optical adhesive film (Applied Biosystems) to prevent evaporation. Each cofactor was incubated with BdcA for 30 min and then subjected to a heat gradient in the presence of SYPRO Orange. The temperature was gradually increased from 25°C to 95°C using a 7900HT Fast Real-Time PCR System (Applied Biosystems). A charge-coupled device detector monitored changes in the intensity of the SYPRO Orange fluorescence. The NADPH and NADP samples exhibited a high initial fluorescence during the assay; however, both samples also exhibited a sharp sigmoidal curve and thus a T_m_ was readily determined. Data were analyzed and T_m_ values computed using the DSF analysis calculation software [22], [23]. Twelve independent experiments were performed.

### Isothermal Titration Calorimetry

Immediately prior to the ITC experiments, SEC (Superdex 75 26/60, GE Healthcare) was used to transfer BdcA into assay buffer followed by concentration of the BdcA protein. Ligands were also dissolved in assay buffer, and both protein and ligand were degassed under vacuum. ITC experiments were performed using a VP-ITC (GE Healthcare) at 25°C. Ligands (600 µM) were titrated (10 µL injections every 200 s, 35 times for all runs except c-di-GMP, in which the time between injections was 300 s) into the cell containing 12 µM monomeric BdcA under constant stirring (307 rpm). Titration of the ligand into buffer alone resulted in a negligible heat of dilution for each ligand. The active protein concentration was adjusted by fitting the data to a one-site binding model. Association constant (K_a_) values were calculated using Microcal Origin 7.0 software.

## Results and Discussion

### BdcA is a dimer

**Figure 1:**
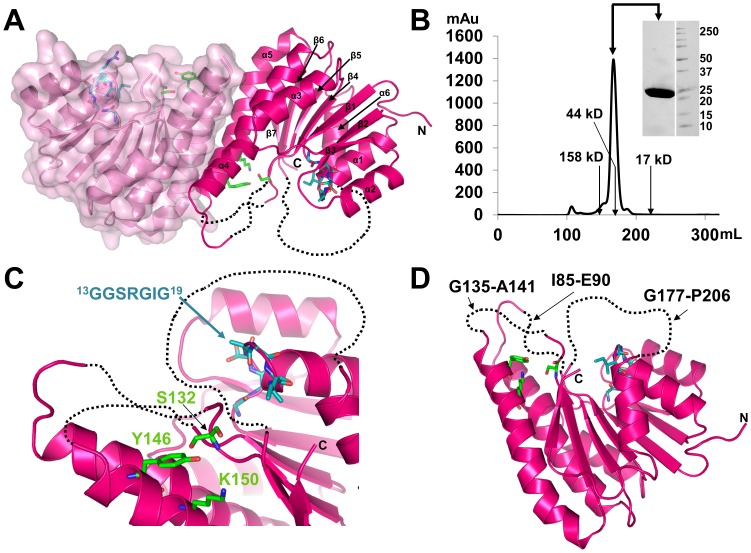
BdcA crystallizes and exists in solution as a dimer. **A**) BdcA dimer, with one monomer colored in dark pink and one in light pink. The glycine-rich cofactor binding motif is depicted as cyan sticks in each monomer and the catalytic triad is shown as green sticks. Disordered loops are displayed as dotted lines. Secondary structural elements are labeled. **B**) Size exclusion chromatogram of BdcA with elution volumes of MW standards indicated (predicted molecular weight of the BdcA monomer is 24.85 kDa). **C**) Close-up of the BdcA catalytic triad (green sticks) and glycine-rich loop (cyan sticks); colored as in (A). **D**) Same as (**C**) except the disordered loops are labeled.

The structure of BdcA was solved using molecular replacement using a poly-Ala version of *R. prowazekii* FabG as a search model (PDB 3F9I) [14]. The final BdcA structure was refined to a resolution of 2.05 Å, resulting in an R_free_ of 22.2% (**Table 1**). Two molecules of BdcA are present in the asymmetric unit and are related by a near perfect two-fold axis (**Figure 1A, 1D**). Each subunit adopts a Rossmann fold composed of a central parallel β-sheet with 7 β-strands sandwiched on both sides by 3 α-helices. The BdcA homodimer is stabilized by a large interface, burying 2132 Å^2^ of surface area and is mediated by multiple hydrophobic residues (Phe103, Ile107, Tyr111, Ala144, Ala145, Ala148, Ala152, Met156 and Leu160) from helices α4 and α5 of each subunit. BcdA is also a dimer in solution, as confirmed using size exclusion chromatography (**Figure 1B**) where it elutes at a volume nearly identical to that of a 44 kDa standard (BdcA monomer MW  = 24.9 kDa, BdcA dimer MW  = 49.8 kDa), and thus the dimer represents the biologically relevant quaternary structure of the protein.

### BdcA is a member of the short-chain dehydrogenase/reductase family

A structure homology search using Dali identified BdcA to be most similar to the short-chain dehydrogenase/reductase (SDR) superfamily of proteins (**Table 2**) [24]. This family of proteins includes over 2000 annotated enzymes and despite the low levels of sequence identity between them, their three-dimensional structures are strikingly similar [25], [26]. SDR proteins typically consist of ∼250 amino acids, are either homo-dimeric or homo-tetrameric and adopt the highly conserved nucleotide-binding Rossmann fold [27]. Tetrameric SDR family members mostly exhibit 222 symmetry, with two of the monomers forming a dimer interface analogous to that identified in BdcA [28]–[30]. This large protein family catalyzes NAD(P)(H)-dependent oxidation/reduction reactions on a wide range of substrates including alcohols, steroids, sugars, aromatic compounds and xenobiotics [26]. More than 1000 structures were identified by DALI to by similar to BdcA, with Z-scores between 27.8 and 11.9. Those identified to be most similar are 3-oxoacyl-(acyl-carrier-protein) reductases and glucose 1-dehydrogenases; the protein with the highest sequence identity (49%) is SM_b20456, a dehydrogenase/reductase from *S. meliloti* 1021 (PDBID 3V2G; **Table 2**).

**Table 2:** BdcA structural homologs as determined by DALI and FFAS.

PDBID	FFAS Score	Z-score	RMSD (Å)	Seq ID (%)	Description
*Top Hits, Dali*					
3GRP	−82.1	27.8	1.5	34	3-OXOACYL-(ACP) REDUCTASE
3F9I*	−85.1	27.8	1.3	40	3-OXOACYL-(ACP) REDUCTASE
3AUS	−80.5	27.1	1.6	33	GLUCOSE 1-DEHYDROGENASE 4
3V2G	−88.7	26.3	1.5	49	3-OXOACYL-(ACP) REDUCTASE
*NADP(H)*					
3F9I*	−85.1	27.8	1.3	40	3-OXOACYL-(ACP) REDUCTASE
3SJ7	−83.9	25.8	1.5	35	3-OXOACYL-(ACP) REDUCTASE
3OSU	–	25.0	1.6	35	3-OXOACYL-(ACP) REDUCTASE
1Q7B	–	25.5	1.8	38	3-OXOACYL-(ACP) REDUCTASE
*NAD(H)*					
2AG5	−84.3	24.3	1.7	27	DEHYDROGENASE/REDUCTASE (SDR) 6

*model used for molecular replacement.

Analysis of previously determined SDR protein structures led to the identification of several cofactor binding motifs that regulate specificity and catalysis. The SDR active site consists of a conserved catalytic triad (Ser-Tyr-Lys), which is C-terminal to the cofactor binding motif, Gly-X_3_-Gly-X-Gly [25], [27]. BdcA adopts the Rossmann fold typical of SDRs and contains the classic dinucleotide-binding motif, Gly13-X_3_-Gly17-X-Gly19 (**Figures 1C, 1D** **,** **2A**). BdcA also contains the active site residues conserved in the SDR family of proteins: Ser132, Tyr146, and Lys150 (located between β5 and α5, **Figures 1C, 1D** **,** **2A, 2B**).

**Figure 2:**
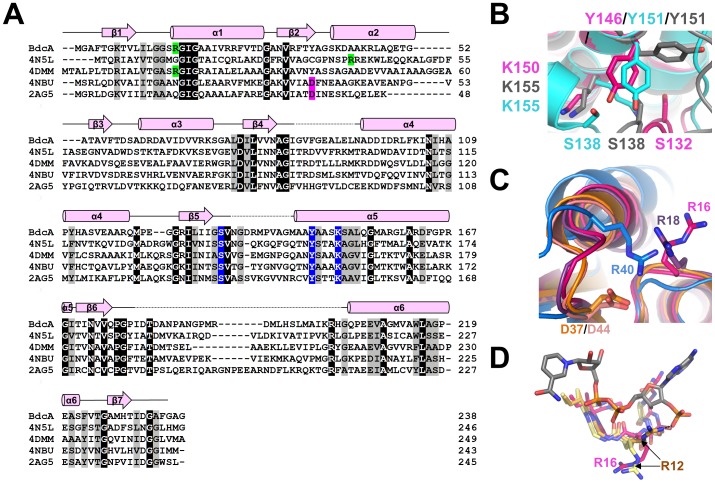
Short-chain dehydrogenase/reductase elements of BdcA. **A**) Sequence alignment of BdcA and homologous SDR members whose cofactors have been identified. Identical amino acids are highlighted in black and similar amino acids are highlighted in gray; α-helices are depicted as cylinders above the sequence alignment and the β-strands as arrows. Disordered loops are displayed as dotted lines. Conserved residues for NADP(H) binding are shown in lime and NAD(H) binding are shown in pink. The catalytic triad is highlighted in blue. **B**) Superposition of the residues that constitute the catalytic triad in BcdA (dark pink) and *E. coli* apo-FabG (gray, PDB:1I01; catalytically incompetent) and *E. coli* FabG bound to NADP+ (cyan, PDB:1Q7B; catalytically competent). **C**) Superposition of SDR family members that bind NADP(H), highlighting cofactor specificity residues. BdcA (dark pink, NADPH-specific), 4N5L (NADPH-specific, blue), 4DMM (NADP-specific, dark purple); all contain basic residues either in the Glycine-rich motif (BdcA, 4DMM) or after β2 (4N5L,). 4NBU (NADH-specific, coral) and 2AG5 (NAD-specific, orange) both contain an aspartic acid directly after the second β-strand. **D**) Predicted re-orientation of BdcA Arg16 upon NADPH binding. Overlay of BdcA (dark pink) with apo-SaFabG1 (yellow, PDB: 3OSU) and SaFabG1:NADPH (gold, PDB: 3SJ7; NADPH is depicted in dark gray).

### BdcA is predicted to bind NADPH

Superposition of BdcA with other SDR proteins bound to their respective cofactors reveals that the BdcA catalytic triad is in a conformation incompatible for catalysis (**Figure 2B**). First, Lys150 is orientated such that the ε-amino group is directed away from Ser132 and Tyr146. In addition, the Cα of Ser132 is shifted between 5.5 to 6.5 Å from the corresponding positions of this Ser in other SDR-cofactor-bound proteins. These shifts are due in part because the three loops that structure the active site in each subunit are presumed to be disordered due to a lack of electron density (subunit A: Gly86-Leu94, Asn134-Gly142, Ile179-His203; subunit B: Ile85-Glu90, Gly135-Ala141, Gly177-Pro206; **Figure 1A, 1D**). The loop with the most missing residues is near the C-terminus, which is also the most variable sequence in SDR enzymes and which is believed to be important for substrate specificity (**Figure 2A**) [26]. In most SDR proteins, the loops surrounding the active site are disordered in the apo-protein and become ordered upon binding cofactor and substrate [31]. Indeed, the same loops are disordered in the protein whose structure is most similar to BdcA, *Bartonella henselae* FabG (PDB 3GRP; **Table 2**) and *Rickettsia prowazekii* FabG (PDB 3F9I; **Table 2**) [14]. In *E. coli* FabG (PDB 1I01), these loops are disordered in the absence of cofactor, but become ordered when bound to NADP+ (PDB 1Q7B, **Table 2**) and orient the catalytic residues in the optimal positions for catalysis (**Figure 2B**) [32]. Thus, most likely cofactor binding to BdcA also positions the active site residues into catalytically competent orientations.

Structural differences have been observed for SDRs depending whether they bind NAD(H) or NADP(H). NAD(H) specificity is defined by an acidic residue directly C-terminal to the second β-strand, approximately 20 amino acids C-terminal of the glycine-rich segment. This residue forms hydrogen bonds to the 2′- and 3′-hydroxyls of the adenine ribose [33]. In contrast, NADP(H)-specific enzymes contain a basic residue within the glycine-rich motif immediately preceding the second conserved glycine, and/or in the loop after the second β-strand. These residue(s) are responsible for binding the 2′-phosphate [26]. In BdcA, the residue preceding the second conserved glycine in the glycine-rich motif is Arg16, suggesting that the endogenous cofactor of BdcA is NADP(H) (**Figure 2A,C**). **Figure 2D** shows a superposition of apo-BdcA with *Staphylococcal* β-ketoacyl-ACP reductase 1 (SaFabG1), the most structurally similar SDR that is bound to NADPH, in both its cofactor bound and unbound state (PDB 3SJ7 and 3OSU, respectively; **Table 2**) [34]. The structurally homologous arginine (Arg12) in SaFabG1 is orientated toward the NADPH molecule in the cofactor bound state, with the arginine side chain forming a hydrogen bond with the pyrophosphate moiety of NADPH. In contrast, in both apo-BdcA and apo-SaFabG1, this arginine side chain is pointed away from the cofactor binding pocket and adopts an unfavorable position for binding. This suggests that the Arg16 side chain in BdcA likely reorients upon cofactor binding, allowing it to engage the NADP(H).

### NADPH is the biologically relevant cofactor of BdcA

**Figure 3:**
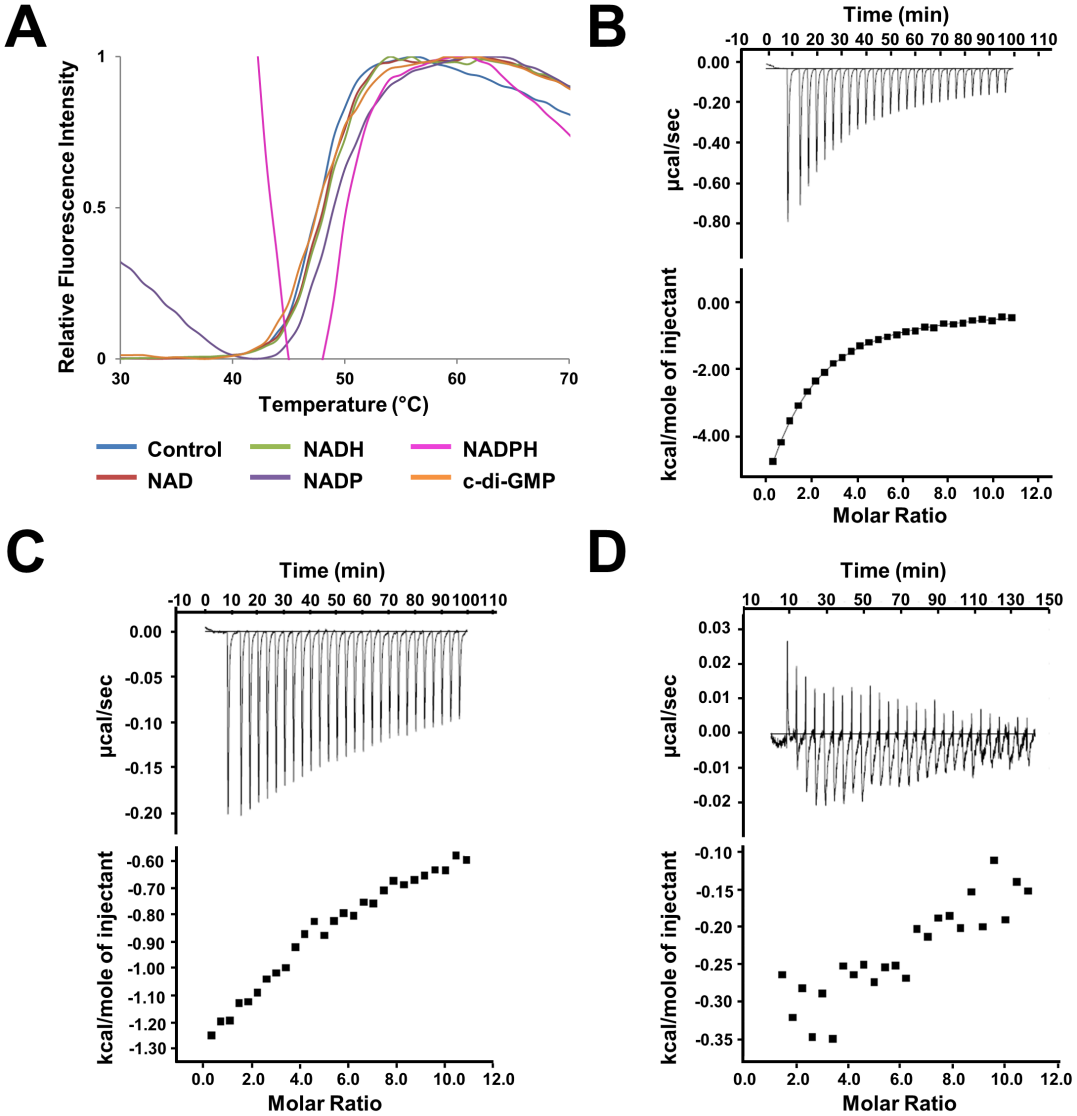
BdcA cofactor is NADPH. **A**) Thermal denaturation curves for BdcA. **B–D**) ITC data for BcdA with different cofactors (**B**, NADPH; **C**, NADP; **D**, c-di-GMP). The raw isothermal titration calorimetry data is shown in the upper panels, whereas the derived binding isotherm plotted against molar ratio of titrant are shown in the lower panels.

To further experimentally confirm that NADP(H) is the biologically relevant cofactor of BdcA, we used both differential scanning fluorimetry (DSF) assays and isothermal titration calorimetry (ITC). We profiled the thermal stability of BdcA in the presence of various cofactors (NAD, NADH, NADP, NADPH or c-di-GMP; **Figure 3A**). The only cofactor that resulted in a significant change in the melting temperature was NADPH, which had a T_m_ of 50.8±0.3°C for NADPH compared to 47.7±0.3°C for the buffer control (**Table 3**). In contrast, the other cofactors resulted in either no change (NAD, NADH or c-di-GMP) or a very weak shift (NADP with a ΔT_m_ = 1.0°C), which correlate with no or very weak binding, respectively (**Table 3**). As NADPH led to the largest change in T_m_, this suggested that NADPH is the likely endogenous cofactor of BdcA.

**Table 3:** Co-factor effects on BdcA thermal stability*.

Cofactor	Tm (°C)	ΔTm (°C)
Buffer	47.7±0.3	–
NAD	47.9±0.3	0.2
NADH	48.1±0.6	0.4
NADP	48.7±0.5	1.0
NADPH	50.8±0.3	3.1
c-di-GMP	47.6±0.5	0.1

*T_m_ shift data: Values represent means ± SD of twelve independent experiments

To confirm this result and quantify the binding affinity of NADPH with BdcA, we used ITC (**Figure 3B–D**). Titration of NADPH into BdcA was performed in triplicate and yielded a typical binding isotherm (**Figure 3B**). NADPH binds to BdcA with a K_d_ of 25.9±4.1 µM (**Table 4**). Despite the abundance of SDR enzymes, only a handful of SDR family members have had their affinities for their respective cofactors characterized using ITC. Interestingly, the observed K_d_ for BdcA with NADPH complex is ∼7–15 times higher than that of other members in the immediate SDR family (SDRvv:NADPH, K_d_ = 3.5 µM; ZmRDH:NAD, K_d_ = 2.72 µM; DHDPR:NADPH, K_d_ = 1.5 µM) [35]–[37]. However, the binding affinity is comparable with other oxidoreductases that are more distantly related (PaGDH:NADH, K_d_ = 18.5 µM; OcDH:NADH: K_d_ = 14 µM) [38], [39]. Titration of NADP into BdcA exhibited an isotherm indicative of even weaker binding (**Figure 3C**) with titration of NAD, NADH and c-di-GMP into BdcA resulting in only heats of dilution; i.e., no binding (**Figure 3D**). Thus, these data again demonstrate that NADPH is the most likely endogenous cofactor of BdcA.

**Table 4:** Thermodynamic and dissociation constants for BdcA:NADPH derived from ITC experiments at 25°C.

Complex	K_d_ (µM)	ΔH (kcal·mol^−1^)	-TΔS (kcal·mol^−1^)
BdcA: NADPH	25.9±4.1	−15.2±2.4	9.0±2.5

Performed in triplicate; values reported are the experimental average and standard deviation.

## Conclusions

The structure of BdcA reveals that it is a member of the short-chain dehydrogenase/reductase family of enzymes. Using two complementary binding assays, we show that BdcA binds specifically to NADPH, and thus is highly likely its endogenous cofactor. Previously, BdcA was hypothesized to suppress biofilm formation by binding and quenching the secondary messenger c-di-GMP [6]. However, we were unable to show binding of c-di-GMP to BdcA using both DSF and ITC, excluding the possibility of a direct interaction. Furthermore, BdcA does not contain any of the well-known c-di-GMP binding motifs present in other proteins (GGDEF, EAL, HD-GYP) [40]. Thus, our data suggests that BdcA most likely influences biofilm dispersal by regulating a process that affects a c-di-GMP related pathway. Previous studies have shown that BdcA also affects biofilm dispersal and is related to the transport of the quorum-sensing (QS) signal autoinducer 2 (AI-2) [6], [41]. Some, albeit few, oxidoreductases have been shown to be involved in quorum sensing related phenotypes [42]–[44]. One example is *B. megaterium* P450BM-3, which has been shown to oxidize acyl homoserine lactones (ASHLs) and acyl homoserines (ASHs), thereby destroying the QS ability of these molecules [43]. Similarly, *P. aeruginosa* BpiB09 has been shown to reduce the primary autoinducer 3-oxo-C_12_-HSL, effectively reducing the QS activity of this molecule. Unlike BdcA, BpiB09 expression reduces motility, and thus these two proteins most likely act on different substrates and exhibit different functions [42]. Our structure of BdcA may prove to be another oxidoreductase involved in quorum sensing. Furthermore, because of its demonstrated role in biofilm dispersal, BdcA also represents an attractive target for dismantling biofilms.
